# Factors associated with patient adherence to medical recommendations during a health crisis

**DOI:** 10.1371/journal.pone.0345375

**Published:** 2026-03-19

**Authors:** Anat Reiner-Benaim, Shimon Amar

**Affiliations:** 1 Department of Epidemiology, Biostatistics and Community Health Sciences, School of Public Health, Faculty of Health Sciences, Ben-Gurion University of the Negev, Be'er-Sheva, Israel; 2 Department of Family Medicine, Faculty of Health Sciences, Ben-Gurion University of the Negev, and Clalit Health Services, Southern District, Be'er-Sheva, Israel; Children's National Hospital, George Washington University, UNITED STATES OF AMERICA

## Abstract

**Introduction:**

Patient non-adherence to medical recommendations is associated with significant economic and health consequences, particularly in chronic diseases. While less studied, similar implications are also observed for short-term antibiotic treatment non-adherence. A global pandemic creates a complex medical reality with the potential to impact patient adherence, and it is of interest to investigate its effect on patient adherence regarding common community-acquired infectious diseases. This study focused on primary care clinic visits with a diagnosis of fever or cough and followed the performance of referred laboratory tests and chest X-rays and the purchase of prescribed antibiotics, as adherence outcomes.

**Methods:**

A comprehensive and unique dataset of clinic visits in Southern Israel was used to assess the impact of the pandemic on patient adherence outcomes. We compared the outcomes between the pandemic period and the preceding and succeeding periods and used multivariate modeling to study the impact of the pandemic period on adherence, subject to patient and visit characteristics.

**Results:**

In total, 609,823 visits were analyzed. Before the pandemic, adherence to throat cultures surpassed that of chest X-ray and referred laboratory tests, and cephalosporins exhibiting higher adherence rates than other antibiotics. Social restrictions and lockdown periods were found to be associated with a decrease in patient adherence. Adherence to referred tests decreased among all patients during social restrictions for complete blood count (Odds Ratio (OR)=0.79, 95%CI = 0.64,0.96), and throat culture (OR=0.64, 95%CI = 0.43,0.93) and among older patients for chest X-ray. Adherence to prescribed antibiotics dropped during social restrictions for penicillin (OR=0.26, 95%CI = 0.17,0.40) and cephalosporins (OR=0.52, 95%CI = 0.32,0.86), but increased for macrolides (OR=2.16, 95%CI = 1.20,3.88). Rural clinics were associated with lower adherence in all outcomes, and visits by phone call were associated with lower adherence, most prominently in throat culture (OR=0.32, 95%CI = 0.26,0.38).

**Conclusion:**

This study offers a unique contribution to our understanding of patient adherence to acute infection management strategies during a pandemic. The findings illuminate the significant impact of uncertainty on patient adherence to diagnostic testing and antibiotic regimens. Understanding these multifaceted influences is essential for improving patient outcomes and optimizing antibiotics use and resource availability during public health crises.

## Introduction

The medical literature is replete with research on adherence to medical treatment, especially in chronic diseases, and less prominently regarding antibiotic therapy [1,2] Adherence, defined as the extent to which a patient's actions coincide with the prescribed treatment regimen/advice, plays a pivotal role in achieving optimal health outcomes [3–5]. Adherence to medical treatment poses a significant challenge both for healthcare providers and patients during routine times [5]. The potential negative consequences of non-adherence such as increased health care costs, substantial worsening of disease and even increased mortality, are amplified during public health crises [6]. Public health crises introduce unprecedented stressors, including resource limitations, shifting healthcare priorities, and rapid changes in treatment guidelines, which likely impacted patient-provider communication, access to care, and the overall healthcare experience [7–9]. Furthermore, public health interventions, such as social distancing and lockdowns, while crucial for disease control, can have unintended consequences on patient routine behavior, including reduced healthcare access, increased anxiety, and disruptions to daily lifestyle habits [10].

Thus, exploring patient adherence to medical recommendations in global health crises is crucial for optimizing public health outcomes and mitigating negative impacts [11,12]. By understanding the complex interplay of the individual, the healthcare provider, and the systematic factors that influence patient behavior, we can develop effective strategies to promote adherence and improve overall health during such challenging times [13,14]. Nevertheless, while the factors influencing adherence to monitoring, balancing, and managing chronic diseases during health crises have been well studied, the specific determinants of adherence in common infectious diseases remain under-explored [12]. While research on chronic disease management during pandemics revealed disruptions to routine care, the complexities of managing common infections, including rapid disease progression, diagnostic challenges, and the need for timely interventions, require a distinct focus [15]. Due to the potential medical consequences of non-adherence or overuse of antibiotics in common infections, particularly during medical crises, understanding adherence in this context is crucial for assessing the broader health and economic implications [16].

The Coronavirus Disease 2019 (COVID-19) pandemic introduced a unique medical reality, characterized by all the above-mentioned challenges [17,18]. A particularly striking illustration of the fluctuating decision-making processes observed among both healthcare professionals and patients during the COVID-19 pandemic was the global variable uptake of COVID-19 vaccines [19–21]. This phenomenon was significantly influenced by prevailing medical uncertainties and the often-contradictory nature of media reporting on the subject. In our previous work, we delved into the responses of primary care physicians (PCPs) to these unique challenging conditions [22]. Following our findings on a decline in referral and prescription rates, the current study investigated the patient's perspective. It aimed to assess patient adherence during the COVID-19 pandemic and identify factors that affect the likelihood of adherence. We hypothesized that patients would exhibit altered adherence behaviors, including changes in their willingness to undergo diagnostic tests and their compliance with prescribed treatment regimens.

The study focused on clinic visits with a diagnosis of fever or cough, which are the two most common infectious complaints [23]. To assess patient adherence, we tracked the completion of recommended laboratory tests, chest X-rays, and the acquisition of prescribed antibiotics following primary care physician visits. We used a large dataset of visits to clinics in Southern Israel to assess the impact of the pandemic on patient adherence outcomes. We compared the outcomes between the pandemic period and the preceding and succeeding periods and used multivariate modeling to study the association of the pandemic period with adherence, subject to patient and visit characteristics.

## Methods

### Study population and design

Data were collected retrospectively from the database of Clalit Health Services (CHS), the largest HMO in Israel, on all visits to a PCP in Southern Israel between January 1, 2018, and December 31, 2022, that resulted in a diagnosis of either cough, fever, or both, in accordance with the International Classification of Diseases (ICD-9).

Data collection Data included patient gender, age, clinic sector, type of visit, referrals, prescriptions, performance of tests and purchase of drugs. Laboratory tests included complete blood count (CBC), c-reactive protein (CRP), and throat culture.

The laboratory tests included were the most frequent and comprised nearly 87% of all tests. Other tests were for coronavirus (6.9%), Epstein-Baar virus (EBV,5.4%), or cytomegalovirus (0.4%). Antibiotic medications included penicillin, cephalosporins and macrolides, defined in accordance with the Anatomical Therapeutic Chemical (ATC) classification.

Adherence was defined as performance of the referred laboratory tests and chest X-ray or purchase of the prescribed antibiotic medication within seven days of the visit, in which the referral or the prescription were issued. This cutoff for defining adherence was selected as it aligns with common clinical practice guidelines [24] for the course of many acute infections. Accordingly, it is expected that the patient will perform the laboratory test or purchase the prescribed medication within this time frame to achieve effective medical care.

Other measures for adherence focus mainly on treatment, including the proportion of days covered (PDC) [25,26], defined as the percentage of days within a specified period that the patient had access to the medication. However, such measures do not objectively reflect a patient's action following the physician's referral or prescription. More precise measures that are often impractical for large-scale retrospective studies include a count of pill intake [27] and self-reported adherence, which is subject to significant recall and social desirability biases [28].

**Fig 1:**
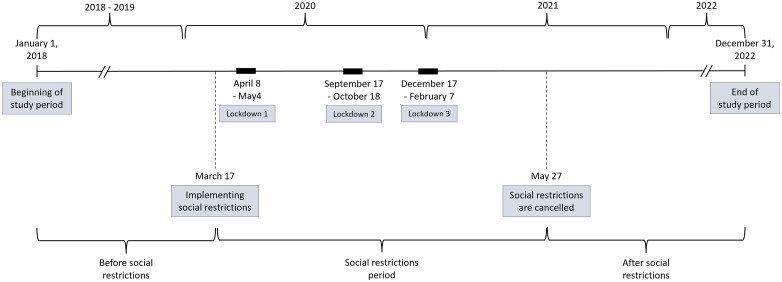
Study period and social restrictions timeline. Including marks of the three lockdowns imposed in Israel during the COVID-19 pandemic.

The data collection period was divided into sub-periods in accordance with the social restrictions imposed in Israel during the COVID-19 pandemic, starting on March 17, 2020, and ending on May 27, 2021, which included social distancing, universal masking and three national lockdowns (Fig 1).

### Ethical considerations

This study was approved by the CHS Research Ethics Committee with a waiver of informed consent given the use of a deidentified data source (study number 0032-21-COM2). All data were fully anonymized by CHS prior to granting access to the researchers. Data was accessed through CHS cloud platform secured by a personal password, temporarily given for the research period only. The study is reported following the Strengthening the Reporting of Observational Studies in Epidemiology (STROBE) reporting guideline for cohort studies. Data was accessed for research purposes on April 9, 2023.

### Statistical analysis

Patient characteristics were compared between social restriction periods using Chi-square test or Fisher's test for categorical variables, analysis of variance for normally distributed variables, and Kruskal-Wallis test for non-normally distributed variables. Normality was tested for continuous variables using the Shapiro–Wilk test. Dichotomous variables were defined for the occurrence of each adherence outcome in a visit. Trend plots marked by restriction periods were used to describe changes in adherence rates across time.

Odds ratios for the effect of each period relative to the period before the restrictions were estimated for each outcome, while adjusting for patient and visit characteristics. A multivariate generalized linear mixed effects model with the binomial family and the logit link function was fit to each adherence outcome. Random effects were used to account for repeated visits of the same patient. Gender, age, clinic sector, visit type, diagnosis, and season at visit were included in each model. We first fit main effects models to consider trends in outcomes across time with high-resolution division of time into periods, such that each lockdown was considered separately. Next, to assess trends that were specific to particular age groups and diagnoses, we fit models that included interaction effects of time period with age and diagnosis, but this time combined all lockdowns as one period, namely the “restrictions period,” to allow useful interpretability of the interaction effects. Adjusted odd-ratios were estimated for the statistically significant interactions to obtain group-specific odds ratios relative to the period before the restrictions. All adjusted interaction odds ratios were visualized on a heatmap across all outcomes and subgroups.

P-values were adjusted for multiple testing by controlling the false discovery rate (FDR) using the Benjamini-Hochberg procedure [29]. Two-sided tests with α = 0.05 were considered statistically significant. To provide a single, intuitive summary measure, we computed an overall adherence index as a visit-weighted means of the component-specific adherence indicators (CBC, CRP, throat culture, chest X-ray, penicillin, cephalosporins, macrolide). Weights equaled the share of each referral/prescription type among all opportunities within the relevant period. This metric complements (but does not replace) the outcome-specific analyses and is reported descriptively.

## Results

### Patients

In total, 609,823 visits to PCP by 216,819 individuals complied with the cohort definitions (Table 1). The mean age across all visits was 22.5 (±26.4) years, and 51.8% of all visits were by females (Table 1). Most visits (69.1%) took place at Jewish urban clinics, compared to 25.7% for Bedouin clinics and 5.2% for Jewish rural clinics. Telehealth visits (by phone call) were 11.5% of all visits. The period before social restrictions lasted 26.5 months, and the period after social restrictions lasted 19.5 months.

**Table 1:** Patient and visit characteristics.

		Total
		(n = 609,823)
**Gender**	**Male**	294,173 (48.2%)
	**Female**	315,650 (51.8%)
**Age at visit (years)**	**mean (SD)**	22.5 (26.4)
	**0-3**	240,367 (39.4%)
	**4-19**	128,357 (21.0%)
	**20-59**	128,357 (24.5%)
	**≥60**	91,524 (15.0%)
**Clinic sector**	**Urban**	421,112 (69.1%)
	**Rural**	31,896 (5.2%)
	**Bedouin**	156,815 (25.7%)
**Visit type**	**Regular**	539,893 (88.5%)
	**Telephone call**	69,930 (11.5%)
**Diagnosis at visit**	**Cough**	292,029 (47.9%)
	**Cough + Fever**	26,188 (4.3%)
	**Fever**	291,606 (47.8%)
**Referral to chest x-ray**		558,463 (8.4%)
*Performed		19,137 (62.7%)
**Visits with a referral to laboratory tests**	**Any test**	510,733 (16.2%)
	**Complete Blood Count**	78,822 (12.9%)
	^**a**^**Performed**	32,581 (58.7%)
	**C-Reactive Protein (CRP)**	21,383 (3.5%)
	^**a**^**Performed**	6,988 (67.3%)
	**Throat Culture**	24,989 (4.1%)
	^**a**^**Performed**	2,169 (91.3%)
**Visits with a drug prescription**	**Any drug**	560,725 (8.1%)
	**Penicillin**	30,867 (5.1%)
	^**a**^**Purchased**	19,975 (35.3%)
	**Cephalosporine**	8,477 (1.4%)
	^**a**^**Purchased**	1,593 (81.2%)
	**Macrolide**	10,134 (1.7%)
	^**a**^**Purchased**	6,222 (38.6%)

^a^Percentage out of all test referrals or prescriptions of the same type.

As shown in Table 2, before and after social restrictions, 39.2%−41.2% of the visits were by children up to three years old, and 14.3%−14.9% were by adults older than 60 years old. During lockdowns, visits of young children were in lower proportions (21.9%−36.2%) and visits of older patients were in higher proportions (17.9%−20.6%). Visits by the intermediate adult age group (ages 20–59) showed a particularly large increase to 30.9%−41.4% during the social restrictions, compared to 23%−23.5% before and after social restrictions. All groups returned to their initial proportions after social restrictions ended.

**Table 2:** Patient and visit characteristics, compared between social restriction periods.

		Before social restrictions	Lockdown 1	Lockdown 2	Lockdown 3	After social restrictions	^b,c^FDR-adjusted p-value
		(n = 283,406)	(n = 4,689)	(n = 7,223)	(n = 11,334)	(n = 217,811)	
**Gender**	**Male**	1,36,894 (48.3%)	2,118 (45.2%)	3489 (48.3%)	5363 (47.3%)	105084 (48.2%)	<0.0001
	**Female**	1,46,512 (51.7%)	2,571 (54.8%)	3734 (51.7%)	5971 (52.7%)	112727 (51.8%)	
**Age at visit (years)**	**mean (SD)**	22.2 (26.4)	32.3 (26.3)	27.3 (26.8)	27.3 (27.6)	21.5 (26.2)	<0.0001
	**0-3**	1,11,133 (39.2%)	1,025 (21.9%)	2,340 (32.4%)	4,108 (36.2%)	89,764 (41.2%)	<0.0001
	**4-19**	63,393 (22.4%)	758 (16.2%)	1,163 (16.1%)	1,604 (14.2%)	46,644 (21.4%)	
	**20-59**	66,582 (23.5%)	1,941 (41.4%)	2,425 (33.6%)	3,507 (30.9%)	50,151 (23.0%)	
	**≥60**	42,298 (14.9%)	965 (20.6%)	1,295 (17.9%)	2,115 (18.7%)	31,252 (14.3%)	
**Clinic sector**	**Urban**	1,95,558 (69.0%)	3,596 (76.7%)	5,096 (70.6%)	8,059 (71.1%)	1,47,972 (67.9%)	<0.0001
	**Rural**	14,556 (5.1%)	282 (6.0%)	386 (5.3%)	539 (4.8%)	11,540 (5.3%)	
	**Bedouin**	73,292 (25.9%)	811 (17.3%)	1,741 (24.1%)	2,736 (24.1%)	58,299 (26.8%)	
**Visit type**	**Regular**	2,74,843 (97.0%)	2,686 (57.3%)	4,584 (63.5%)	8,457 (74.6%)	1,84,796 (84.8%)	<0.0001
	**Telephone call**	8,563 (3.0%)	2,003 (42.7%)	2,639 (36.5%)	2,877 (25.4%)	33,015 (15.2%)	
**Diagnosis at visit**	**Cough**	1,37,432 (48.5%)	2,642 (56.3%)	3,167 (43.8%)	6,546 (57.8%)	1,00,690 (46.2%)	<0.0001
	**Cough + Fever**	12,251 (4.3%)	207 (4.4%)	286 (4.0%)	423 (3.7%)	9,918 (4.6%)	
	**Fever**	1,33,723 (47.2%)	1,840 (39.2%)	3,770 (52.2%)	4,365 (38.5%)	1,07,203 (49.2%)	
**Referral to chest x-ray**		2,58,167 (8.9%)	4,283 (8.7%)	6,780 (6.1%)	10,370 (8.5%)	1,99,918 (8.2%)	<0.0001
*Performed		8,661 (65.7%)	138 (66.0%)	151 (65.9%)	322 (66.6%)	7,601 (57.5%)	<0.0001
**Visits with a referral to laboratory tests**	**Any test**	2,33,834 (17.5%)	4,200 (10.4%)	6,388 (11.6%)	9,806 (13.5%)	1,83,433 (15.8%)	<0.0001
	**Complete Blood Count**	38,847 (13.7%)	431 (9.2%)	721 (10.0%)	1,312 (11.6%)	27,189 (12.5%)	<0.0001
	^**a**^**Performed**	15,892 (59.1%)	202 (53.1%)	301 (58.3%)	527 (59.8%)	11,268 (58.6%)	0.0090
	**C-Reactive Protein (CRP)**	9,554 (3.4%)	130 (2.8%)	211 (2.9%)	325 (2.9%)	8,262 (3.8%)	<0.0001
	^**a**^**Performed**	3,063 (67.9%)	51 (60.8%)	69 (67.3%)	99 (69.5%)	2,737 (66.9%)	0.2716
	**Throat Culture**	13,434 (4.7%)	65 (1.4%)	137 (1.9%)	275 (2.4%)	8,646 (4.0%)	<0.0001
	^**a**^**Performed**	1,051 (92.2%)	12 (81.5%)	13 (90.5%)	35 (87.3%)	791 (90.9%)	<0.0001
**Visits with a drug prescription**	**Any drug**	2,57,077 (9.3%)	4,448 (5.1%)	6,783 (6.1%)	10,663 (5.9%)	2,01,747 (7.4%)	<0.0001
	**Penicillin**	16,157 (5.7%)	119 (2.5%)	267 (3.7%)	393 (3.5%)	10,745 (4.9%)	<0.0001
	^**a**^**Purchased**	11,228 (30.5%)	94 (21.0%)	241 (9.7%)	332 (15.5%)	5,453 (49.3%)	<0.0001
	**Cephalosporine**	4,643 (1.6%)	72 (1.5%)	78 (1.1%)	144 (1.3%)	2,401 (1.1%)	<0.0001
	^**a**^**Purchased**	836 (82.0%)	15 (79.2%)	28 (64.1%)	35 (75.7%)	422 (82.4%)	<0.0001
	**Macrolide**	5,780 (2.0%)	52 (1.1%)	97 (1.3%)	138 (1.2%)	2,996 (1.4%)	<0.0001
	^**a**^**Purchased**	3,187 (44.9%)	22 (57.7%)	57 (41.2%)	77 (44.2%)	2,266 (24.4%)	<0.0001

^a^Percentage out of all test referrals or prescriptions of the same type.

^b^Comparisons were made using Chi-square test or Fisher's test for categorical variables, analysis of variance for normally distributed variables, and Kruskal-Wallis test for non-normally distributed variables.

^c^P-values were adjusted for multiple testing to control the FDR (False Discovery Rate), using the BH procedure.

During lockdown periods, between 25% and 42.7% of the visits were telehealth visits. After social restrictions, such visits became more common (15%) compared to their rate before the restrictions (3%). Throughout all periods, around 4% of the visits included diagnosis of both cough and fever, with the rest of the visits divided similarly between visits with only cough diagnosis and visits with only fever diagnosis. While visits with only cough diagnosis and visits with only fever diagnosis were typically similar in proportion, which ranged between 46.2% and 49.2%, the first and third lockdowns were characterized by a shift towards more visits with only cough diagnosis (56.3%−57.8%), while the second lockdown was characterized with a shift towards visits with only fever diagnosis (52.2%).

### Adherence outcomes

As shown in Table 1, 8.4% of the visits resulted in a referral to a chest X-ray, 16.2% of the visits resulted in a referral to laboratory tests, and 8.1% of the visits resulted in prescribing antibiotic drugs. Complete blood count was the most frequently referred laboratory test (12.9%) and penicillin was the most frequently prescribed antibiotic drug (5.1%). We studied the associations of these referrals and prescriptions with the pandemic period in our previous paper [22].

**Fig 2:**
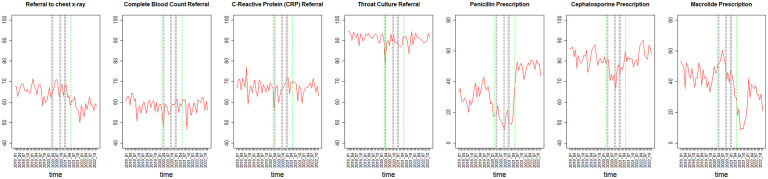
Patient adherence rates (%) across time (monthly). Includes marks of beginning and end of social restrictions period (dotted green lines) and the three lockdown periods (grey). Adherence to laboratory test referrals showed sporadic drops once social restrictions started. Adherence dropped for CBC, CRP, and throat culture. Adherence to penicillin and cephalosporins dropped during the second lockdown but increased after social restrictions. Adherence to macrolides increased during the first lockdown but dropped during the next lockdowns.

Changes in patient adherence rates during the pandemic period varied between outcomes (Table 2 and Fig 2). Adherence to laboratory test referrals showed sporadic drops once social restrictions started. The performance rate dropped from 59.1% to 53.1% for CBC, from 67.9% to 60.8% for CRP, and from 92.2% to 81.5% for throat culture. Nevertheless, the performance rate of chest X-ray did not decrease during the social restrictions, remaining between 65.7% and 66.6%, yet it dropped during the period after social restrictions to a level as low as 50% (Fig 2).

In contrast to the referred tests, adherence to antibiotic prescriptions was more substantially changed during the pandemic period. The Purchase rate of penicillin showed a major drop from 30.5% before the social restrictions to as low as 9.7% during the second lockdown, and an increase to as high as 50%, well beyond the initial levels, after social restrictions (Fig 2). A similar trend was observed for cephalosporins, which dropped from 82% adherence before the restrictions to 64.1% during the second lockdown, and increased to 82.4%, nearly the original level, after social restrictions.

A different trend was observed for macrolides, as their purchase rate increased to 57.7% during the first lockdown, compared to 44.9% before the restrictions. However, during the next lockdowns adherence dropped to near the original levels (41.2%−44.2%). Interestingly, the decline continued after the social restrictions ended, reaching a new nadir point of 10% (Fig 2).

Overall adherence was calculated as a weighted average of adherence rates across all outcomes, with weights proportional to the number of referrals or prescriptions issued for each outcome. While before social restrictions, the overall adherence rate was 45%, during social restrictions it declined to 40% during the first lockdown, dropped to 21% during the second lockdown, and then increased to 32%. After social restrictions ended, it rose to a peak of 51%.

### Factors affecting adherence during and after the pandemic

The adjusted odds ratios provided precise measures for trends in patient adherence, in comparison to the period before the restrictions, while accounting for all patient and visit characteristics. Full models result, including odds ratios calculated for interactions with age and diagnosis, are provided within S1 and S2 Tables.

**Fig 3:**
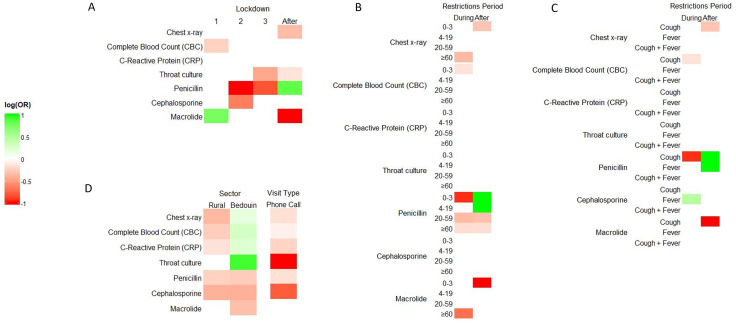
Heatmap of adjusted odds ratios (aOR) with 95% confidence intervals (CI) for patient adherence, relative to the period before social restrictions. Adjusted odds ratios are presented on a log scale, centered at zero. Red indicates a decrease, while green indicates an increase. aOR were estimated by multivariate generalized linear mixed effects models with the binomial family and logit link. A. aOR during each lockdown and after social restrictions, relative to the period before the restrictions, adjusted for gender, age, clinic sector, visit type, diagnosis, and season at visit (see also Table 3). B-C. aOR during and after social restrictions, stratified by age (B) and by diagnosis (C), calculated based on the interaction effect with period, with adjustment for all other covariates. D. aOR for rural and Bedouin clinics, relative to urban clinics (left panel) and for telehealth visits (by phone call), relative to in-person visits. Full models’ results are provided in Supplementary S1 and S2 Tables, including regression coefficients, aOR and 95% CI.

**Fig 4:**
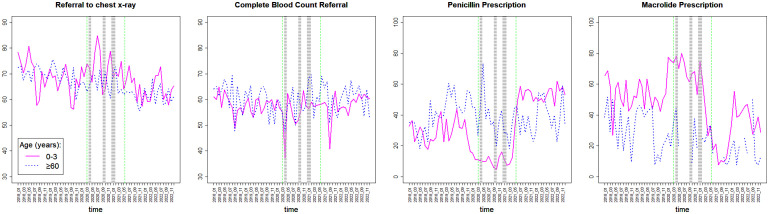
Patient adherence rates (%) across time (monthly) for the youngest and oldest age groups. For chest X-ray, adherence decreased during social restrictions for patients aged 60 years or older and decreased after social restrictions for patients aged 0-3 years. For CBC referrals, adherence decreased during the first lockdown for patients of ages 0-3. For penicillin prescription, adherence decreased during social restrictions and increased afterwards for children aged 0-3. Opposite trends were observed for macrolides.

Odds ratios by subgroups are presented in Fig 3 and corresponding age-specific trends in adherence are shown by Fig 4. For chest X-ray, adherence decreased during social restrictions for patients of age 60 or older (OR=0.70, S2 Table and Fig 3B) and further decreased after social restrictions (OR=0.71, Table 3 and Fig 3A), most prominently for patients of ages 0–3 (Figs 3B and 4) with only cough diagnosis (Fig 3C). For CBC test, adherence decreased during the first lockdown (OR=0.79, Table 3 and Fig 3A), most prominently for patients of ages 0–3 (Figs 3B and 4) with only cough diagnosis (Fig 3C) and returned to previous levels afterwards. For throat culture, adherence decreased during the third lockdown and onward (OR=0.64, Table 3 and Fig 3A).

**Table 3:** Adjusted odds ratios for time-period effect on patient adherence^a^.

Patient Adherence		Period		Odds Ratio (95% CI)	FDR-adjusted p-value
**Peformance of chest x-ray**	**Chest x-ray**	lockdown 1		1.10 (0.89, 1.37)	0.4520
		lockdown 2		1.11 (0.90, 1.37)	0.3975
		lockdown 3		1.15 (0.99, 1.32)	0.1017
		after social restrictions		0.71 (0.68, 0.74)	<0.0001
		between lockdowns		1.00 (0.94, 1.06)	0.9460
		Clinic sector	Rural	0.70 (0.65, 0.75)	<0.0001
			Bedouin	1.19 (1.13, 1.26)	<0.0001
		Visit type	Telephone call	0.85 (0.79, 0.91)	<0.0001
**Performance of laboratory tests**	**Complete Blood Count (CBC)**	lockdown 1		0.79 (0.64, 0.96)	0.0334
		lockdown 2		0.91 (0.78, 1.06)	0.2982
		lockdown 3		1.07 (0.95, 1.21)	0.3050
		after social restrictions		0.97 (0.94, 1.01)	0.1685
		between lockdowns		0.93 (0.89, 0.98)	0.0091
		Clinic sector	Rural	0.77 (0.73, 0.82)	<0.0001
			Bedouin	1.34 (1.29, 1.39)	<0.0001
		Visit type	Telephone call	0.92 (0.87, 0.99)	0.0333
	**C-Reactive Protein (CRP)**	lockdown 1		0.72 (0.49, 1.04)	0.1298
		lockdown 2		0.93 (0.69, 1.26)	0.7041
		lockdown 3		1.10 (0.85, 1.41)	0.5546
		after social restrictions		0.96 (0.90, 1.02)	0.2679
		between lockdowns		0.93 (0.85, 1.03)	0.2214
		Clinic sector	Rural	0.86 (0.75, 0.98)	0.0335
			Bedouin	1.26 (1.16, 1.36)	<0.0001
		Visit type	Telephone call	0.80 (0.71, 0.90)	0.0004
	**Throat Culture**	lockdown 1		0.55 (0.28, 1.08)	0.1298
		lockdown 2		0.81 (0.44, 1.47)	0.5548
		lockdown 3		0.64 (0.43, 0.93)	0.0338
		after social restrictions		0.87 (0.79, 0.96)	0.0139
		between lockdowns		0.79 (0.68, 0.92)	0.0060
		Clinic sector	Rural	0.94 (0.80, 1.09)	0.4854
			Bedouin	2.53 (2.11, 3.04)	<0.0001
		Visit type	Telephone call	0.32 (0.26, 0.38)	<0.0001
**Purchase of drugs**	**Penicillin**	lockdown 1		0.60 (0.38, 0.95)	0.0500
		lockdown 2		0.26 (0.17, 0.40)	<0.0001
		lockdown 3		0.44 (0.33, 0.58)	<0.0001
		after social restrictions		2.35 (2.23, 2.49)	<0.0001
		between lockdowns		0.48 (0.43, 0.53)	<0.0001
		Clinic sector	Rural	0.79 (0.68, 0.91)	0.0024
			Bedouin	0.76 (0.72, 0.81)	<0.0001
		Visit type	Telephone call	0.85 (0.74, 0.97)	0.0321
	**Cephalosporine**	lockdown 1		0.98 (0.54, 1.80)	0.9675
		lockdown 2		0.52 (0.32, 0.86)	0.0191
		lockdown 3		0.91 (0.60, 1.38)	0.7290
		after social restrictions		1.11 (0.97, 1.28)	0.1822
		between lockdowns		0.87 (0.73, 1.04)	0.1746
		Clinic sector	Rural	0.67 (0.54, 0.83)	0.0007
			Bedouin	0.66 (0.57, 0.77)	<0.0001
		Visit type	Telephone call	0.45 (0.38, 0.54)	<0.0001
	**Macrolide**	lockdown 1		2.16 (1.20, 3.88)	0.0189
		lockdown 2		0.87 (0.57, 1.33)	0.5795
		lockdown 3		0.95 (0.66, 1.36)	0.8220
		after social restrictions		0.33 (0.29, 0.36)	<0.0001
		between lockdowns		0.88 (0.76, 1.01)	0.1112
		Clinic sector	Rural	1.01 (0.80, 1.28)	0.9415
			Bedouin	0.72 (0.65, 0.79)	<0.0001
		Visit type	Telephone call	1.20 (0.95, 1.52)	0.1724

^a^Adjusted odds ratios (aOR) with 95% confidence intervals (CI) for time-period effect on patient adherence.

Odds ratios are relative to the preceding period, clinic sectors are relative to urban clinics, and visit types are relative to regular visits.

aOR were estimated by multivariate generalized linear mixed-effects models with the binomial family and logit link, adjusted for gender, age, clinic sector, visit type, diagnosis, and season at visit. Full model results are provided within S1 Table.

### FDR – False discovery rate

### CBC – Complete blood count

The odds ratios for patient adherence to purchasing prescribed antibiotic treatments further clarified the opposite trends between the different types of drugs. Adherence decreased for penicillin during the restrictions (OR of 0.26–0.44, Table 3 and Fig 3A), most prominently for children of ages 0–3 (Figs 3B and 4) for only cough diagnosis (Fig 3C). It also decreased for cephalosporins during the second lockdown (OR=0.52, Table 3 and Fig 3A). In contrast, purchase of macrolides increased substantially during the first lockdown (OR=2.16, Table 3 and Fig 3A), except for patients of ages 60 or older (Figs 3B and 4).

After social restrictions, the opposite trends for penicillin and macrolides were further observed, as adherence for penicillin increased substantially (OR=2.35, Table 3 and Fig 3A), particularly for children (Figs 3B and 4) with cough or fever (Fig 3C), while adherence to macrolides decreased (OR=0.33, Table 3 and Fig 3A), particularly among children of age 0–3 (Fig 3B and 4) with cough only (Fig 3C).

Clinic sector (Jewish urban, Jewish rural or Bedouin) and visit type (regular or by phone call) were found to impact patient adherence (Table 3 and Fig 3D). Visits to rural clinics were associated with lower adherence in most outcomes (OR of 0.67–0.94, Fig 3D), and visits to Bedouin clinics were associated with higher adherence in all test outcomes (Fig 3D, left panel), particularly throat culture (OR=2.53), and lower adherence in drug purchase (OR of 0.66–0.76). Telehealth visits (by phone call) were associated with lower adherence to most outcomes (Fig 3D, right panel), most prominently throat culture (OR=0.32) and cephalosporins (OR=0.45), but also chest X-ray (OR=0.85), CBC (OR=0.92), CRP (OR=0.80) and penicillin (OR=0.85).

## Discussion

This large-scale retrospective cohort study investigated patient adherence, as defined by response to diagnostic tests referrals and to antibiotic prescriptions. It compared adherence patterns prior to the COVID-19 pandemic period with those observed during and after social restrictions and lockdowns.

Analyzing patient adherence to ancillary tests and antibiotic treatments during the 26.5 months preceding the COVID-19 pandemic revealed varying adherence rates. Adherence to throat cultures was highest, followed by chest X-rays, then CRP levels, and finally, CBC. A similar hierarchy was observed for antibiotic treatments, with cephalosporins exhibiting higher adherence rates than macrolides and penicillin.

Continuous analysis of adherence during the following 33.5 months, encompassing the COVID-19 pandemic and the period succeeding it, revealed decreased adherence during social restrictions. This decline was particularly notable for chest X-rays among patients older than 60, CBC among children aged 0–3, and throat cultures across all age groups. Adherence to penicillin regimens, especially among children aged 0–3, also decreased during restrictions, while cephalosporins adherence initially decreased and subsequently rebounded, reaching new peaks after restrictions were ended. Conversely, macrolide prescription adherence increased during restrictions and decreased afterward. Lower adherence rates were associated with visits to rural and Bedouin clinics, as well as phone consultations.

The COVID-19 pandemic significantly impacted medication adherence and healthcare services for patients with chronic conditions. Studies examining non-adherence among patients with chronic non-communicable diseases during the pandemic identified psychological, social, and environmental factors [6]. Impairments in mobility, freedom of movement, social connections, limited access to healthcare facilities, fear of infection, and disruptions in medication supply chains significantly impacted the ability to maintain a balance in those diseases, thereby requiring interventions across multiple medical and behavioral domains [13]. In Ethiopia, a study found that 72% of diabetic and hypertensive patients had poor medication adherence during the pandemic, with 57% reporting negative impacts on follow-up visits, medication availability, or affordability [30]. Conversely, a Korean study showed improved medication adherence and persistence among patients with dyslipidemia during the pandemic, with an increased proportion of days covered and lower risks of medication discontinuation [31].

Patient adherence is a multifaceted behavior influenced by a complex interplay of factors, both in routine and emergency settings. Under normal circumstances, non-adherence is associated with determinants such as forgetfulness, accessibility issues, concerns about side effects, perceived treatment inefficacy, low health literacy, stigmatization, lifestyle choices, financial constraints, psychological factors, inadequate social support, and suboptimal physician-patient communication [32] However, during public health emergencies like the COVID-19 pandemic, these challenges are exacerbated and compounded by additional stressors. Disruptions in medication supply chains, economic hardships, restricted access to healthcare services, heightened fear and anxiety, and communication barriers become prominent factors affecting adherence. Therefore, interventions aimed at improving patient adherence must consider both the chronic determinants and the acute challenges posed by emergency situations, ensuring tailored strategies that address the unique needs of individuals in diverse contexts [12,33].

The aforementioned factors may help elucidate our findings, starting with those observed before the pandemic. Patients’ perception of illness severity, coupled with their confidence in the diagnosis and treatment, led to higher adherence rates for broad-spectrum antibiotics, such as cephalosporins and macrolides, that are often prescribed for more severe infections, compared to penicillin [34,35]. Considerations of convenience and accessibility explain the higher compliance rates for throat cultures, performed in the doctor's office, compared to chest X-rays and blood tests, which typically involve less availability, more effort on the patient's part, or discomfort, such as in blood tests [36]. Conversely, when symptoms are more significant, such as fever and cough, and medical evaluation is better communicated, there will be higher adherence to diagnostic tests and treatment [37].

The global medical uncertainty that predominated during the early stages of the COVID-19 pandemic led in part to empirical decision-making, both in diagnosis and treatment. Common symptoms of typical infectious diseases also aligned with those of COVID-19. In our previous work [22], we discussed the impact of COVID-19 on physicians’ decisions, decreasing both prescribed antibiotics and ancillary tests [38,39]. The pandemic exacerbated existing barriers to healthcare access, particularly for vulnerable populations. Factors such as convenience, support systems and perceived risk influenced patients’ decisions to seek care, leading to decreased adherence to medical recommendations, especially among the elderly and young children. In addition, patients were influenced by media reports. For instance, macrolides were briefly perceived as effective in inhibiting inflammatory processes in COVID-19, which may explain the increased patient adherence to this treatment [40,41]. Adherence to throat culture and penicillin and cephalosporins treatments was deemed unnecessary due to the increasing incidence of COVID-19 and the fact that sore throat was one of its symptoms.

Following the lockdowns and restrictions, our initial study [23], observed changes in infectious disease patterns, with a corresponding rise in common illnesses such as pharyngitis. Indeed, in the current study we found a significant increase in patient adherence to cephalosporins and penicillin treatment during the post-restrictions period and return to nearly pre-restrictions levels in adherence to throat culture. This further highlights the interpretation that in the absence of active epidemic and conflicting medical information being disseminated to the public, regardless of accuracy, the public is more likely to heed medical recommendations, even for relatively minor infectious diseases.

Furthermore, we observed “delayed effects” of treatment adherence during the post-restrictions period. For instance, adherence to macrolides decreased significantly, possibly due to lingering negative perceptions of the treatment arising from the pandemic period, when conflicting messages were circulated regarding its efficacy versus its contribution to antibiotic resistance. Similar behavior was observed in the public's perception of chest X-rays being ineffective in diagnosing the cause of a cough, as was the case during the pandemic. We can venture to speculate that these negative memories will fade over time, and future adherence will return to pre-pandemic levels.

Despite the active patient involvement in decision-making, we believe it does not reflect a lack of trust in primary care but results from a period of heightened uncertainty causing low health literacy, along suboptimal physician-patient communication [42]. This claim is supported by our finding of difference in adherence between in-person and telemedicine consultations, and of consultation rates and adherence to most tests and treatments returning to their original levels in the post-pandemic period. We can speculate that the media played a significant role in shaping public perception of the pandemic, which in turn influenced patient behavior and treatment decisions, as well as subconsciously during physician-patient interactions in a period of heightened uncertainty. Patients made informed decisions based on the information they had accumulated at that time. Fortunately, at the end of the period, no increases in complications or mortality were observed in the study population, which may question the real need of some regimens given in first place, but not future resistant microbial strains [38,39,43]. If this is the case, we can positively leverage the influence of the media on patient decision-making and treatment adherence in the future [44].

Nevertheless, the media's dissemination of unsubstantiated information during times of crisis influenced public perception of effective treatments [45]. Amidst the scientific and medical uncertainty surrounding the pandemic, the public was bombarded with claims regarding the efficacy of various interventions, including macrolides, vitamins, steroids, and turmeric. Despite the lack of rigorous scientific evidence, these claims gained traction, particularly among those experiencing fear and helplessness. Consequently, it proved challenging to dissuade the public from pursuing these unproven treatments, which often relied on the placebo effect. The absence of readily available, official, and expert-vetted information during crises creates a void that is naturally filled by rumors and unverified claims. This gap between the public's need for knowledge and the lack of credible sources exacerbates the problem of misinformation. When individuals are anxious and uncertain, they are more susceptible to believing in unsubstantiated claims, particularly when these claims offer hope or a sense of control [17,46].

Our study revealed marked deviations in antibiotic adherence within the youngest (0–3 years) and oldest patient cohorts during the COVID-19 pandemic, reflecting a confluence of age-specific vulnerabilities. For young children, adherence is inherently dependent on caregiver actions, which were likely influenced by heightened pandemic-related anxieties, disruptions to routine care, and potential hesitancy toward medical interventions amid widespread misinformation [47]. Caregivers may have faced an increased burden due to childcare closures and economic strain, impacting their ability to adhere to medication regimens. In contrast, older adults, who often manage multiple comorbidities, were likely to encounter barriers related to reduced mobility, fear of infection at healthcare facilities, and potential cognitive decline, further compounded by social isolation during lockdowns [48]. Moreover, both groups are susceptible to communication challenges, with young children relying on caregivers’ interpretation of medical advice and older adults potentially experiencing difficulties in understanding or remembering instructions. These findings align with broader research indicating that pandemic-induced disruptions disproportionately affected vulnerable populations, leading to reduced healthcare utilization and adherence to prescribed treatments [49].

As noted, visits to rural and Bedouin clinics, as well as phone-call consultations, were associated with lower adherence rates. During pandemics, when uncertainty is high and conventional medical treatments may seem inadequate, some rural and Bedouin populations may exhibit a heightened reliance on traditional healing practices. This trend is particularly pronounced in conservative communities where belief in traditional knowledge is deeply rooted. Limited access to healthcare due to long distances, transportation challenges, inflexible clinic hours, and socioeconomic factors such as poverty and low health literacy can also complicate adherence. While telehealth offers potential benefits, its implementation in these communities presents new challenges, including communication barriers and language disparities, potentially further distancing these populations from conventional healthcare [50]. Consequently, trust in official information erodes, adherence to guidelines diminishes, and existing health disparities widen. It is crucial to note that these phenomena are not uniform across all rural and Bedouin communities. Significant variations exist among different groups, influenced by their unique sociocultural and economic characteristics, especially during times of crisis. Understanding the cultural, psychological, and socioeconomic dimensions of this phenomenon is essential for developing effective intervention strategies [51,52].

A proportion of the non-adherence observed during restrictions likely reflects contextual constraints (financial, geographic, organizational, and information-related) rather than individual preferences alone. We therefore interpret sector- and visit-type differences as indicators of structural barriers to access and communication during crisis conditions, not solely as behavioral choices.

## Strengths and limitations

Our study has several notable strengths. First, it leverages a large and diverse cohort of more than 600,000 primary-care visits across pre-pandemic, pandemic, and post-pandemic periods, providing high statistical power and representation of vulnerable subgroups such as young children, older adults, rural residents, and Bedouin populations. Second, the use of comprehensive electronic health records from a single health system ensured consistent capture of referrals, diagnostic test performance, and antibiotic purchases, thereby minimizing information bias [22,23]. Third, the application of multivariable mixed-effects models with interaction terms enabled evaluation of heterogeneous effects across patient subgroups while accounting for repeated visits [29].

At the same time, several limitations should be acknowledged. First, as an observational study, our analyses cannot establish causal relationships and should be interpreted as associations. Second, we lacked contextual variables such as comorbidities, socioeconomic status, or access-to-care indicators, leaving potential residual confounding [6,12,13]. Third, adherence was approximated by completion of tests and medication purchase, which may not fully reflect ingestion or therapeutic compliance [53]. Fourth, some care episodes may have occurred outside the primary-care setting, though the requirement for physician-issued antibiotic prescriptions in Israel reduces the likelihood of missing large numbers of antibiotic purchases. Finally, while our findings are robust within the Israeli healthcare system, their generalizability to other healthcare systems remains to be established [30,31,33].

In conclusion, this study offers a unique contribution to our understanding of patient adherence to acute infection management strategies during a pandemic, specifically COVID-19. Examining a large and diverse population over five years, we identified significant variations in adherence to both diagnostic testing and antibiotic treatment, influenced by the evolving pandemic landscape, prevailing disease patterns, and associated medical uncertainty [54].

These findings have critical implications for both antibiotic stewardship and pandemic preparedness [26,38,55]. Our results underscore the need for tailored interventions to address the specific challenges faced by diverse populations, including children, older adults, and cultural groups such as Bedouins and residents of rural communities. Such interventions may include stricter medical criteria for prescribing and dispensing certain antibiotics during pandemics, implemented for limited durations and based on accumulating evidence. Critically, effective communication is essential, encompassing both national-level public health messaging and clear, consistent communication between healthcare teams and their patients. Well-informed and confident medical professionals can effectively convey scientific evidence to patients, who are generally receptive to their guidance. Robust communication strategies are therefore crucial for addressing patient concerns, combating misinformation, and improving adherence during periods of heightened anxiety [56].

Furthermore, this study highlights the potential for digital health interventions, such as mobile health applications, to support adherence tracking and personalized care during public health crises [54]. Future research should also explore adherence among vulnerable populations, including immuno-compromised patients, individuals with disabilities, and those with chronic complicated diseases. Comparative studies with other countries are necessary to assess the generalizability of these findings and inform global pandemic preparedness and response strategies.

In periods of increasing uncertainty, more effort is warranted to promote better adherence to therapy, especially when patients try to self-manage their disease. Our results further highlight the relationship between patient preference and adherence, the complex nature of adherence and the need for adequate patient education. In light of our findings, actionable recommendations for improving adherence in future crises [57] may include implementing patient reminders for test completions, enhancing telehealth platforms for better communication, and adjusting public health campaigns to address specific misconceptions and sub-populations.

## Supporting information

S1 Table Main effects models. An excel table that includes regression coefficients and odds ratios for main effects models, with 95% confidence limits and FDR-adjusted p-values, for each adherence outcome.
(CSV)

S2 Table Main effects and interaction effects models. An Excel table that includes regression coefficients and odds ratios for main effects and interaction effects models, with 95% confidence limits and FDR-adjusted p-values, for each adherence outcome.
(CSV)

## References

[1] Losi S, Berra CCF, Fornengo R, Pitocco D, Biricolti G, Federici MO. The role of patient preferences in adherence to treatment in chronic disease: a narrative review. Drug Target Insights. 2021;15:13–20. doi: 10.33393/dti.2021.2342 34785884 PMC8591552

[2] West LM, Cordina M. Educational intervention to enhance adherence to short-term use of antibiotics. Res Social Adm Pharm. 2019;15(2):193–201. doi: 10.1016/j.sapharm.2018.04.011 29685459

[3] Sluijs E, Dulmen S, Dijk L, Ridder D, Heerdink RR, Bensing J. Patient adherence to medical treatment: a meta review. 2006. 10.1186/1472-6963-7-55 PMC1955829 17439645

[4] Bissonnette JM. Adherence: a concept analysis. J Adv Nurs. 2008;63(6):634–43. doi: 10.1111/j.1365-2648.2008.04745.x 18808585

[5] Burnier M. The role of adherence in patients with chronic diseases. Eur J Intern Med. 2024;119:1–5. doi: 10.1016/j.ejim.2023.07.008 37479633

[6] Hassan S-U-N, Zahra A, Parveen N, Khatoon F, Bangi NA, Hosseinzadeh H. Quality of Life and Adherence to Healthcare Services During the COVID-19 Pandemic: A Cross-Sectional Analysis. Patient Prefer Adherence. 2022;16:2533–42. doi: 10.2147/PPA.S378245 36147381 PMC9488595

[7] Souvatzi E, Katsikidou M, Arvaniti A, Plakias S, Tsiakiri A, Samakouri M. Trust in Healthcare, Medical Mistrust, and Health Outcomes in Times of Health Crisis: A Narrative Review. Societies. 2024;14(12):269. doi: 10.3390/soc14120269

[8] Filip R, Gheorghita Puscaselu R, Anchidin-Norocel L, Dimian M, Savage WK. Global Challenges to Public Health Care Systems during the COVID-19 Pandemic: A Review of Pandemic Measures and Problems. J Pers Med. 2022;12(8):1295. doi: 10.3390/jpm12081295 36013244 PMC9409667

[9] Zheng Y, Liu J, Tang PK, Hu H, Ung COL. A systematic review of self-medication practice during the COVID-19 pandemic: implications for pharmacy practice in supporting public health measures. Front Public Health. 2023;11:1184882. doi: 10.3389/fpubh.2023.1184882 37397709 PMC10310324

[10] Hills S, Eraso Y. Factors associated with non-adherence to social distancing rules during the COVID-19 pandemic: a logistic regression analysis. BMC Public Health. 2021;21(1):352. doi: 10.1186/s12889-021-10379-7 33581734 PMC7881344

[11] van Dulmen S, Sluijs E, van Dijk L, de Ridder D, Heerdink R, Bensing J. Patient adherence to medical treatment: a review of reviews. BMC Health Serv Res. 2007;7:55. doi: 10.1186/1472-6963-7-55 17439645 PMC1955829

[12] Ruksakulpiwat S, Zhou W, Niyomyart A, Wang T, Kudlowitz A. How does the COVID-19 pandemic impact medication adherence of patients with chronic disease?: A systematic review. Chronic Illn. 2023;19(3):495–513. doi: 10.1177/17423953221110151 35971949 PMC9382573

[13] Olmastroni E, Galimberti F, Tragni E, Catapano AL, Casula M. Impact of COVID-19 Pandemic on Adherence to Chronic Therapies: A Systematic Review. Int J Environ Res Public Health. 2023;20(5):3825. doi: 10.3390/ijerph20053825 36900831 PMC10001666

[14] Stewart S-JF, Moon Z, Horne R. Medication nonadherence: health impact, prevalence, correlates and interventions. Psychol Health. 2023;38(6):726–65. doi: 10.1080/08870446.2022.2144923 36448201

[15] Cantón R, Akova M, Langfeld K, Torumkuney D. Relevance of the Consensus Principles for Appropriate Antibiotic Prescribing in 2022. J Antimicrob Chemother. 2022;77(Suppl_1):i2–9. doi: 10.1093/jac/dkac211 36065724 PMC9445850

[16] Almomani BA, Hijazi BM, Awwad O, Khasawneh RA. Prevalence and predictors of non-adherence to short-term antibiotics: A population-based survey. PLoS One. 2022;17(5):e0268285. doi: 10.1371/journal.pone.0268285 35588114 PMC9119442

[17] Sheek-Hussein M, Abu-Zidan FM, Stip E. Disaster management of the psychological impact of the COVID-19 pandemic. Int J Emerg Med. 2021;14(1):19. doi: 10.1186/s12245-021-00342-z 33761863 PMC7988636

[18] Vindegaard N, Benros ME. COVID-19 pandemic and mental health consequences: Systematic review of the current evidence. Brain Behav Immun. 2020;89:531–42. doi: 10.1016/j.bbi.2020.05.048 32485289 PMC7260522

[19] Sallam M. COVID-19 Vaccine Hesitancy Worldwide: A Concise Systematic Review of Vaccine Acceptance Rates. Vaccines. 2021;9:160. 33669441 10.3390/vaccines9020160 PMC7920465

[20] Galanis P, Vraka I, Katsiroumpa A, Siskou O, Konstantakopoulou O, Katsoulas T, et al. COVID-19 Vaccine Uptake among Healthcare Workers: A Systematic Review and Meta-Analysis. Vaccines (Basel). 2022;10(10):1637. doi: 10.3390/vaccines10101637 36298502 PMC9610263

[21] Berliner Senderey A, Ohana R, Perchik S, Erev I, Balicer R. Encouraging Uptake of the COVID-19 Vaccine Through Behaviorally Informed Interventions: National Real-World Evidence From Israel. SSRN Journal. 2021. doi: 10.2139/ssrn.3852345

[22] Reiner-Benaim A, Amar S. Trends in decision-making by primary care physicians regarding common infectious complaints. Infect Dis (Lond). 2024;56(8):644–52. doi: 10.1080/23744235.2024.2344795 38647537

[23] Amar S, Avni YS, O’Rourke N, Michael T. Prevalence of Common Infectious Diseases After COVID-19 Vaccination and Easing of Pandemic Restrictions in Israel. JAMA Netw Open. 2022;5(2):e2146175. doi: 10.1001/jamanetworkopen.2021.46175 35103792 PMC8808334

[24] Almomani BA, Hijazi BM, Al-Husein BA, Oqal M, Al-Natour LM. Adherence and utilization of short-term antibiotics: Randomized controlled study. PLoS One. 2023;18(9):e0291050. doi: 10.1371/journal.pone.0291050 37669277 PMC10479900

[25] Prieto-Merino D, Mulick A, Armstrong C, Hoult H, Fawcett S, Eliasson L, et al. Estimating proportion of days covered (PDC) using real-world online medicine suppliers’ datasets. J Pharm Policy Pract. 2021;14(1):113. doi: 10.1186/s40545-021-00385-w 34965882 PMC8715592

[26] Baryakova TH, Pogostin BH, Langer R, McHugh KJ. Overcoming barriers to patient adherence: the case for developing innovative drug delivery systems. Nat Rev Drug Discov. 2023;22(5):387–409. doi: 10.1038/s41573-023-00670-0 36973491 PMC10041531

[27] Tedla YG, Bautista LE. Factors associated with false-positive self-reported adherence to antihypertensive drugs. J Hum Hypertens. 2017;31(5):320–6. doi: 10.1038/jhh.2016.80 27853149 PMC6062205

[28] Stirratt MJ, Dunbar-Jacob J, Crane HM, Simoni JM, Czajkowski S, Hilliard ME, et al. Self-report measures of medication adherence behavior: recommendations on optimal use. Transl Behav Med. 2015;5(4):470–82. doi: 10.1007/s13142-015-0315-2 26622919 PMC4656225

[29] Benjamini Y, Hochberg Y. Controlling the False Discovery Rate: A Practical and Powerful Approach to Multiple Testing. Journal of the Royal Statistical Society Series B: Statistical Methodology. 1995;57(1):289–300. doi: 10.1111/j.2517-6161.1995.tb02031.x

[30] Shimels T, Asrat Kassu R, Bogale G, Bekele M, Getnet M, Getachew A, et al. Magnitude and associated factors of poor medication adherence among diabetic and hypertensive patients visiting public health facilities in Ethiopia during the COVID-19 pandemic. PLoS One. 2021;16(4):e0249222. doi: 10.1371/journal.pone.0249222 33822807 PMC8023457

[31] Jang J, Oh HJ, Lee E-K. Despite the pandemic: upward trajectories of medication adherence and persistence in patients with dyslipidemia. Front Pharmacol. 2024;15:1488452. doi: 10.3389/fphar.2024.1488452 39712500 PMC11658976

[32] DiMatteo MR, Haskard-Zolnierek KB, Martin LR. Improving patient adherence: a three-factor model to guide practice. Health Psychology Review. 2012;6(1):74–91. doi: 10.1080/17437199.2010.537592

[33] Kretchy IA, Asiedu-Danso M, Kretchy J-P. Medication management and adherence during the COVID-19 pandemic: Perspectives and experiences from low-and middle-income countries. Res Social Adm Pharm. 2021;17(1):2023–6. doi: 10.1016/j.sapharm.2020.04.007 32307319 PMC7158799

[34] Lee JS, Giesler DL, Gellad WF, Fine MJ. Antibiotic Therapy for Adults Hospitalized With Community-Acquired Pneumonia: A Systematic Review. JAMA. 2016;315(6):593–602. doi: 10.1001/jama.2016.0115 26864413

[35] Casey JR, Pichichero ME. Meta-analysis of cephalosporins versus penicillin for treatment of group A streptococcal tonsillopharyngitis in adults. Clin Infect Dis. 2004;38(11):1526–34. doi: 10.1086/392496 15156437

[36] Zhao C, Ma W. Patient resistance towards clinicians’ diagnostic test-taking advice and its management in Chinese outpatient clinic interaction. Soc Sci Med. 2020;258:113041. doi: 10.1016/j.socscimed.2020.113041 32480183

[37] Haag M, Hersberger KE, Arnet I. Assessing Medication Adherence Barriers to Short-Term Oral Antibiotic Treatment in Primary Care-Development and Validation of a Self-Report Questionnaire (BIOTICA). Int J Environ Res Public Health. 2021;18(15):7768. doi: 10.3390/ijerph18157768 34360062 PMC8345617

[38] Oliveira I, Rego C, Semedo G, Gomes D, Figueiras A, Roque F, et al. Systematic Review on the Impact of Guidelines Adherence on Antibiotic Prescription in Respiratory Infections. Antibiotics (Basel). 2020;9(9):546. doi: 10.3390/antibiotics9090546 32867122 PMC7557871

[39] Rocha V, Estrela M, Neto V, Roque F, Figueiras A, Herdeiro MT. Educational Interventions to Reduce Prescription and Dispensing of Antibiotics in Primary Care: A Systematic Review of Economic Impact. Antibiotics (Basel). 2022;11(9):1186. doi: 10.3390/antibiotics11091186 36139965 PMC9495011

[40] Crawford-Faucher A. Antibiotics for the Treatment of COVID-19. Am Fam Physician. 2022;105(3):237–8. 35289561

[41] Rashad A, Nafady A, Hassan MH, Mansour H, Taya U, Bazeed SES, et al. Therapeutic efficacy of macrolides in management of patients with mild COVID-19. Sci Rep. 2021;11(1):16361. doi: 10.1038/s41598-021-95900-z 34381155 PMC8357809

[42] Hyvert S, Yailian A-L, Haesebaert J, Vignot E, Chapurlat R, Dussart C, et al. Association between health literacy and medication adherence in chronic diseases: a recent systematic review. Int J Clin Pharm. 2023;45(1):38–51. doi: 10.1007/s11096-022-01470-z 36369411

[43] Chan Y-H, Fan MM, Fok C-M, Lok ZL, Ni M, Sin C-F, et al. Antibiotics nonadherence and knowledge in a community with the world’s leading prevalence of antibiotics resistance: implications for public health intervention. Am J Infect Control. 2012;40(2):113–7. doi: 10.1016/j.ajic.2011.03.017 21741119 PMC7115258

[44] Williams SN, Dienes K, Jaheed J, Wardman JK, Petts J. Effectiveness of communications in enhancing adherence to public health behavioural interventions: a COVID-19 evidence review. Philos Trans A Math Phys Eng Sci. 2023;381(2257):20230129. doi: 10.1098/rsta.2023.0129 37611630 PMC10446905

[45] Chan HF, Brumpton M, Macintyre A, Arapoc J, Savage DA, Skali A, et al. How confidence in health care systems affects mobility and compliance during the COVID-19 pandemic. PLoS One. 2020;15(10):e0240644. doi: 10.1371/journal.pone.0240644 33057450 PMC7561184

[46] Sule S, DaCosta MC, DeCou E, Gilson C, Wallace K, Goff SL. Communication of COVID-19 Misinformation on Social Media by Physicians in the US. JAMA Netw Open. 2023;6(8):e2328928. doi: 10.1001/jamanetworkopen.2023.28928 37581886 PMC10427940

[47] Araújo LAd, Veloso CF, Souza MdC, Azevedo JMCd, Tarro G. The potential impact of the COVID-19 pandemic on child growth and development: a systematic review. J Pediatr (Rio J). 2021;97:369–77. 32980318 10.1016/j.jped.2020.08.008 PMC7510529

[48] Daniali SS, Rahimi M, Salarvand S. Age discrimination in delivery of health services to old people during COVID-19 pandemic: a scoping review study. JGG. 2022;70(1):68–82. doi: 10.36150/2499-6564-n415

[49] Dalton M, Sanderson B, Robinson LJ, Homer CSE, Pomat W, Danchin M, et al. Impact of COVID-19 on routine childhood immunisations in low- and middle-income countries: A scoping review. PLOS Glob Public Health. 2023;3(8):e0002268. doi: 10.1371/journal.pgph.0002268 37611014 PMC10446229

[50] Butzner M, Cuffee Y. Telehealth interventions and outcomes across rural communities in the United States: narrative review. J Med Internet Res. 2021;23:e29575. 10.2196/29575 PMC8430850 34435965

[51] Rahman MdM, Rahman MdA, Siddique MdKB, Islam MdR, Rahim MM ur. Impact of COVID-19 on Essential Health Care of Rural People in Northern Bangladesh: A Cross-Sectional Study. Int J Transl Med Res Public Health. 2023;7(1). doi: 10.21106/ijtmrph.410

[52] Abu-Freha N, Alsana H, El-Saied S, Azbarga Z, Aloka M, Goda T, et al. COVID-19 Vaccination Among the Arab Bedouin Population: Lessons Learned From a Minority Population. Int J Public Health. 2022;67:1604133. doi: 10.3389/ijph.2022.1604133 35392540 PMC8980216

[53] Llor C, Hernández S, Bayona C, Moragas A, Sierra N, Hernández M, et al. A study of adherence to antibiotic treatment in ambulatory respiratory infections. Int J Infect Dis. 2013;17(3):e168-72. doi: 10.1016/j.ijid.2012.09.012 23116609

[54] Kardas P, Lewek P, Matyjaszczyk M. Determinants of patient adherence: a review of systematic reviews. Front Pharmacol. 2013;4:91. doi: 10.3389/fphar.2013.00091 23898295 PMC3722478

[55] Cutler DM, Everett W. Thinking outside the pillbox--medication adherence as a priority for health care reform. N Engl J Med. 2010;362(17):1553–5. doi: 10.1056/NEJMp1002305 20375400

[56] Talie Fenta E, Bogale EK, Anagaw TF. The role of social media on COVID-19 preventive behaviors worldwide, systematic review. PLoS One. 2024;19(7):e0306284. doi: 10.1371/journal.pone.0306284 38985700 PMC11236194

[57] Rincón Uribe FA, Godinho RC de S, Machado MAS, Oliveira KR da SG, Neira Espejo CA, de Sousa NCV, et al. Health knowledge, health behaviors and attitudes during pandemic emergencies: A systematic review. PLoS One. 2021;16(9):e0256731. doi: 10.1371/journal.pone.0256731 34492047 PMC8423234

